# Short-Term Outcomes of the First-Session Prone Position in Patients With Severe Coronavirus Disease 2019: A Retrospective Chart Review

**DOI:** 10.7759/cureus.35437

**Published:** 2023-02-24

**Authors:** Makoto Onji, Shinji Kakizoe, Asuka Nakai, Kanami Shimizu, Yosuke Masui, Koichi Naito, Hironori Mikumo

**Affiliations:** 1 Department of Rehabilitation, Kitakyushu Municipal Medical Center, Kitakyushu, JPN; 2 Department of Nursing, Kitakyushu Municipal Medical Center, Kitakyushu, JPN; 3 Faculty of Medical Science, Nagoya Women’s University, Nagoya, JPN; 4 Department of Respiratory Medicine, Kitakyushu Municipal Medical Center, Kitakyushu, JPN

**Keywords:** covid-19, coronavirus disease 2019, activities of daily living, short-term outcomes, mechanical ventilation, prone position, severe coronavirus disease 2019

## Abstract

Introduction

Prone positioning during ventilation is recommended for patients with severe coronavirus disease 2019 (COVID-19). However, the efficacy of first-session prone positioning in improving short-term outcomes remains unclear. Therefore, we aimed to investigate the impact of the rate of change in partial pressure of oxygen/fraction of inspired oxygen (P/F) ratio before and after initial prone positioning on activities of daily living (ADL) and outcomes at discharge.

Methods

In this retrospective chart review, 22 patients with severe COVID-19 who required ventilator management between April and September 2021 were analyzed. Patients with an improvement in the P/F ratio (after initial prone positioning, compared to that before the session) by > 16mHg and < 16mmHg were defined as responders and non-responders, respectively.

Results

Compared with non-responders, responders had a significantly shorter ventilator duration, a higher Barthel Index at discharge, and a higher proportion of discharged patients. There was a significant between-group difference in chronic respiratory comorbidities, with one case (7.7%) among responders and six cases (66.7%) among non-responders.

Conclusions

This study is the first of its kind to investigate short-term outcomes in patients with COVID-19 requiring ventilator management after initial prone positioning. After initial prone positioning, responders had higher P/F ratios as well as improved ADLs and outcomes at discharge.

## Introduction

Patients with severe acute respiratory syndrome coronavirus 2 (SARS-CoV-2) infection (coronavirus disease 2019 (COVID-19)) experience acute respiratory failure soon after the onset of dyspnea and hypoxemia, generally meeting the criteria for acute respiratory distress syndrome (ARDS) [1]. Treatment options for ARDS include oxygen therapy, ventilatory therapy, and extracorporeal membrane oxygenation (ECMO), as well as antiviral, anti-inflammatory, and anticoagulant medications. Furthermore, it is recommended that patients be placed in the prone position for 12-16 hours per day as an adjunctive treatment [2], depending on their condition, to help improve oxygenation, ventilation-perfusion ratio mismatch, and hemodynamic stability [3]. Early implementation of the prone position in non-COVID-19 patients with ARDS is associated with reduced mortality [4]. Additionally, prone positioning can reduce ventral-dorsal transpulmonary pressure differences and lung compression by the heart [5] and diaphragm, as well as improve the partial pressure of oxygen/fraction of inspired oxygen (P/F) ratio [6]. Currently, prone positioning is actively implemented in >70% of critically ill patients [3].

Several studies have demonstrated the effectiveness of awake-prone positioning under high-flow nasal cannula therapy [7,8]. Intubated patients with COVID-19 present with more severe symptoms. Further, most reports on prone positioning in patients with COVID-19 have focused on physiological changes [9,10], muscle strength and activity after intensive care unit (ICU) discharge [11], and mortality [3]. This study focused on the responsiveness to first-session prone positioning. Several studies have investigated the rate of change in the P/F ratio during the first-session prone positioning; however, they only investigated physiological parameters [9,10] and not muscle strength or activities of daily living (ADLs). Further, studies focusing on muscle strength and activity levels after ICU discharge applied supine positioning as the control [11]. Moreover, alpha and delta variants were rampant [12] in Japan during this period, leading to an increased number of severe patients. In Japan's aging society, the loss of muscle strength and ADLs due to the treatment of severe COVID-19 is a major concern. Currently, the responsiveness of patients to first-session prone positioning and its impact on short-term outcomes remain unclear. Therefore, it is important to examine the short-term effects of first-session prone positioning during early weaning from ventilation in patients with severe COVID-19 requiring ventilator management. This study aimed to investigate the impact of the rate of change in the P/F ratio before and after first-session prone positioning on ADLs and outcomes at discharge.

## Materials and methods

Research design and sampling method

**Figure 1:**
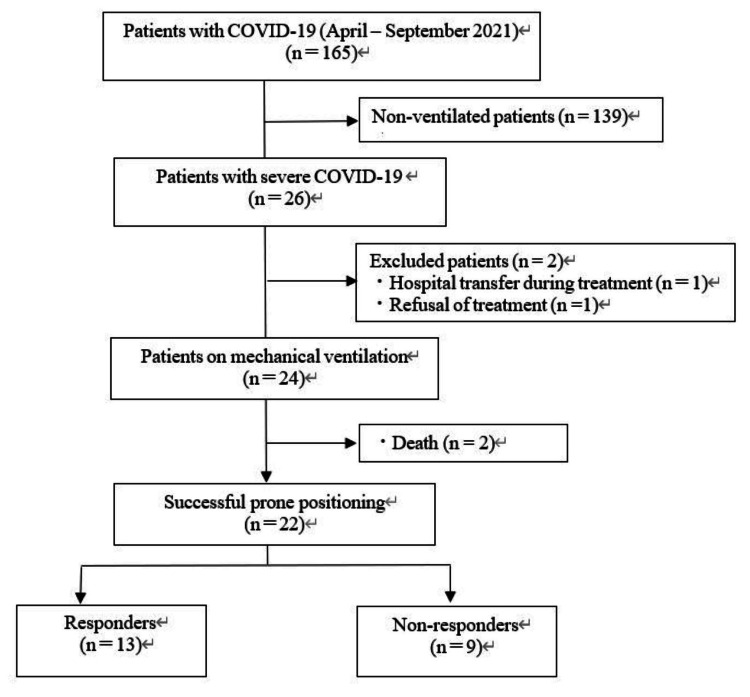
Patient selection flow diagram

This retrospective chart review study included Japanese subjects at a single center (Kitakyushu Municipal Medical Center, Japan). The medical records of 26 patients diagnosed with COVID-19 by polymerase chain reaction testing and placed on ventilator management between April and September 2021 were reviewed, considering CT scans and respiratory symptoms. Among them, four patients were excluded, including two patients who died during hospitalization, one who refused treatment, and one who was suddenly transferred to another hospital for ECMO support. Accordingly, 22 cases were included in the final analysis (Figure 1). Patients who died were excluded from the analysis as they could not be placed in the prone position on admission due to poor hemodynamics.

Measurement items

The primary outcome was the Barthel Index (BI) [13] after extubation and at discharge. BI ranged from fully independent (100 points) to fully assisted (0 points), determined according to the subject's movement status. Age, sex, body mass index (BMI), Charlson Comorbidity Index, and type of comorbidity (chronic respiratory disease, cerebrovascular disease, cardiac disease, diabetes, hypertension, cancer, and obesity) were evaluated as basic characteristics. The P/F ratio in each position and its rate of change, ventilator settings (fraction of inspired oxygen (FiO2) and positive end-expiratory pressure (PEEP)), intubation duration, number of days from onset to intubation, number of days in the hospital after extubation, the total number of days in the hospital, and blood data (lactate dehydrogenase, serum albumin, hemoglobin, and C-reactive protein levels) were evaluated as treatment-related factors. Other rehabilitation-related factors included the Medical Research Council (MRC) sum score at post-extubation and the presence of frailty at discharge. Obesity was defined as a BMI ≥ 28 kg/m2 [14]. Frailty was defined as a Clinical Frailty Scale [15] score ≥ 4.

Definition of P/F ratio parameters and responders

At our hospital, arterial blood gas measurements are routinely taken before the start of prone positioning, two hours, six hours, and 14 hours after the start of prone positioning, and after prone positioning. We evaluated the following parameters: (1) the P/F ratio in the supine position before the first-session prone position (supine position 1: SP1); (2) the highest P/F ratio value in the prone position (prone position: PP); and (3) the P/F ratio in the supine position after the first-session prone position (supine position 2: SP2). Responders were defined as those with a P/F ratio improvement of ≥16 mmHg from SP1 to SP2 [9].

Statistical analyses

Data are presented as the median (interquartile range), regardless of the normality of the distribution. For continuous variables, the unpaired t-test and Mann-Whitney U test were used to compare normally and non-normally distributed variables, respectively. For categorical variables, the chi-squared test was used. Statistical significance was set at P < 0.05. All statistical analyses were performed using EZR on R Commander version 1.55 (Saitama Medical Center, Jichi Medical University, Saitama, Japan) [16].

Introduction of the prone position

The prone position was applied at the discretion of the attending physician, based on a P/F ratio of <200 mmHg immediately after ventilator management, blood test results, CT findings, and treatment response. Sedation was adjusted to achieve a Richmond Agitation-Sedation Scale score of -5 during management in the prone position. The procedure for changing the patient’s position to a prone position at our hospital was as follows. All routes, including ventilators, arterial lines, and intravenous routes, were placed on the same side. A towel large enough to cover the whole body was placed under the patient. Five staff were deployed, including one at the patient's head and two on each side. The staff member at the patient’s head secured the intubation tube and the patient's face while the other four staff members repositioned the patient.

First, the patient’s body was moved sideways to the side opposite the routes. Subsequently, the patient was repositioned to the side-lying position. The patient’s upper body was lifted slightly; further, the upper limbs, which were on the underside of the body, were pulled to the dorsal side. Next, the body was rolled over to a prone position to complete the positioning. To prevent adverse events, including pressure ulcers related to the supine position, we checked for nerve compression and wrinkles in the clothing. Depending on the pathology, the prone position was held for 16-18 hours, from the evening until the following morning.

Ethical considerations

In accordance with the Declaration of Helsinki, an opt-out option was provided on our website, and the study outline was open to the public to allow patients to refuse inclusion in the study. The latest guidelines in Japan allow the use of clinical information for observational studies using an opt-out model [17]. This study was approved by the Institutional Review Board of Kitakyushu Municipal Medical Center (approval number: 202110004).

## Results

Basic characteristics

Table 1 shows the basic demographic characteristics of the 22 patients. The median age was 58 years. The proportions of males and females were 54.5% and 45.5%, respectively. The median BMI was 25.5 kg/m2. The most common comorbidity was obesity (36.4%), followed by chronic respiratory disease (31.8%). There were no significant differences in age, sex, and BMI between responders and non-responders. There was a significant between-group difference in comorbidities, with chronic respiratory disease in one patient (7.7%) in the responder group and six patients (66.7%) in the non-responder group (p = 0.003). Additionally, the proportion of patients with obesity was higher among responders than among non-responders (53.8% vs. 11.1%, p = 0.074).

**Table 1:** Basic characteristics of the patients Note: Values are reported as the median (interquartile range) or the number of patients (percentage).

	Overall (n = 22)	Responders (n = 13)	Non-responders (n = 9)	P-values
Age, years	58 (49–66)	53 (46–64)	61 (53–66)	0.216
Male/female, n (%)	12 (54.5)/10 (45.5)	6 (46.2)/7 (53.8)	6 (66.7)/3 (33.3)	0.415
BMI (kg/m^2^), n (%)	25.5 (22.6–31.7)	29.4 (22.1–34.9)	25.2 (24.2–25.6)	0.186
<18.5	3 (13.6)	1 (7.7)	2 (22.2)	
18.5–25	6 (27.3)	4 (30.8)	2 (22.2)	
25–30	6 (27.3)	2 (15.4)	4 (44.4)	
30–35	5 (22.7)	4 (30.8)	1 (11.1)	
˃35	2 (9.1)	2 (15.4)	0 (0)	
Obesity (BMI > 28 kg/m^2^) (%)	8 (36.4)	7 (53.8)	1 (11.1)	0.074
Charlson Comorbidity Index, n (%)				1
Low	13 (59.1)	7 (53.8)	6 (66.7)	
Medium	8 (36.4)	5 (38.5)	3 (33.3)	
High	1 (4.5)	1 (7.7)	0 (0)	
Comorbidity, n (%)	32	15	17	
Respiratory disease	7 (31.8)	1 (7.7)	6 (66.7)	0.003
Cerebrovascular disease	1 (4.5)	0 (0)	1 (11.1)	0.219
Heart disease	1 (4.5)	0 (0)	1 (11.1)	0.219
Diabetes	6 (27.3)	2 (15.4)	4 (44.4)	0.131
Hypertension	6 (27.3)	3 (23.1)	3 (33.3)	0.592
Cancer	3 (13.6)	2 (15.4)	1 (11.1)	0.771
Length of time between COVID-19 diagnosis and intubation, days	9 (40.9)	10 (10–13)	9 (7–11)	0.079
Number of times in prone position, times	3.5 (3–6)	3 (3–4)	6 (3–7)	0.086
Mechanical ventilation, days	7 (7–10.5)	7 (5–8)	11 (7–12)	0.035
Length of hospital stay after extubation, days	16 (14–21)	16 (15–21)	16 (14–25)	0.867
Total length of hospital stay, days	24 (21–35)	24 (21–28)	28 (21–49)	0.547

Rate of change in the P/F ratio and ventilator settings

**Figure 2:**
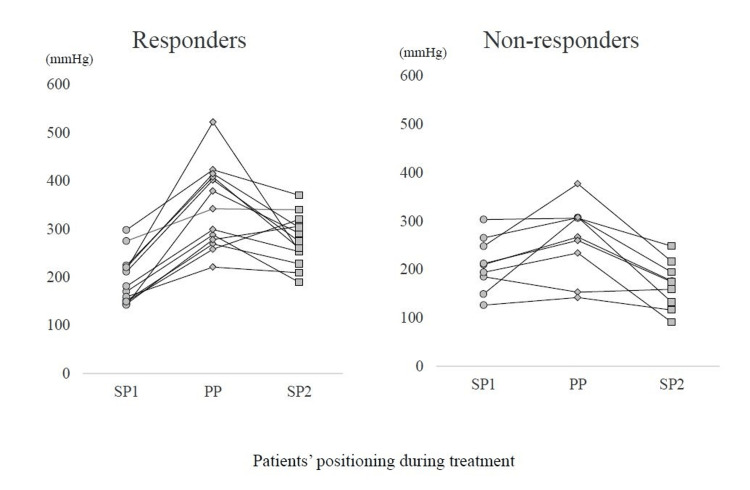
Comparison of the change in the P/F ratio between responders and non-responders P/F: partial pressure of oxygen/fraction of inspired oxygen; SP1 and SP2: supine position before and after prone positioning, respectively; PP: prone positioning.

Figure 2 and Table 2 show the changes in the P/F ratio and ventilator settings. There were significant between-group differences in the PP and SP2 P/F ratios (p = 0.028 and p < 0.01, respectively), with greater values among responders than among non-responders. For all postures, there were no significant between-group differences in the FiO2 and PEEP. There were significant between-group differences in the percent change in the P/F ratio for SP1 → PP and SP1 → SP2 (p = 0.007 and p < 0.01, respectively), with greater values in responders than in non-responders.

**Table 2:** Comparisons in the change in the P/F ratio and ventilator settings according to posture Notes: Values are reported as the median (interquartile range). P/F: partial pressure of oxygen/fraction of inspired oxygen; FiO2: fraction of inspired oxygen; SP1 and SP2: supine position before and after prone positioning, respectively; PP: prone positioning; PEEP: positive end-expiratory pressure.

	Overall (n = 22)	Responders (n = 13)	Non-responders (n = 9)	P-values
P/F ratio (mmHg)				
SP1	202 (153–223)	181 (151–220)	210 (185–248)	0.616
FiO_2 _	0.58 (0.41–0.6)	0.50 (0.4–0.6)	0.55 (0.5–0.6)	0.813
PEEP (cmH_2_O)	11 (10–12)	10 (10–12)	12 (10 –12)	0.411
PP	302 (261–379)	342 (278–408)	267 (234–307)	0.028
FiO_2 _	0.52 (0.4–0.6)	0.45 (0.4–0.6)	0.55 (0.4–0.6)	0.513
PEEP (cmH_2_O)	11 (10–12)	10 (10–12)	12 (10–12)	0.625
SP2	238 (179–286)	274 (253–306)	174 (132–194)	<0.01
FiO_2_	0.45 (0.4–0.55)	0.45 (0.35–0.5)	0.5 (0.4–0.55)	0.252
PEEP (cmH_2_O)	11 (10–12)	10 (10–12)	12 (10–12)	0.540
Change in P/F ratio (%)				
SP1 → PP	58.5 (24.8 to 87.3)	78 (65 to 91)	23 (16 to 49)	0.007
PP → SP2	-25.1 (-35.8 to -13.2)	-15.4 (-31.8 to -5.4)	-34.1 (-42.7 to -18.9)	0.070
SP1 → SP2	16.9 (-13.8 to 36.1)	31.4 (23.6 to 51.0)	-16.2 (-18.2 to -12.9)	<0.01

Comparisons in treatment-related and rehabilitation-related factors

Treatment- and rehabilitation-related factors are shown in Table 3. Responders had a significantly shorter ventilator duration (seven days (5-8) vs. 11 days (7-12), p = 0.035), higher BI at discharge (100 points (90-100) vs. 70 points (40-95), p = 0.029), and a higher proportion of discharged patients (92.3% vs. 33.3%, p = 0.006) compared to that in non-responders.

**Table 3:** Comparisons of clinical, laboratory, and rehabilitation-related factors between responders and non-responders Notes: Values are reported as the median (interquartile range) or the number of patients (percentage). CRP: C-reactive protein; LDH: lactate dehydrogenase; Hb: hemoglobin; Alb: serum albumin; MRC: Medical Research Council sum score.

	Overall (n = 22)	Responders (n = 13)	Non-responders (n = 9)	P-values
Barthel Index				
After extubation	7.5 (0–25)	15 (0–25)	0 (0–15)	0.439
At hospital discharge	95 (70–100)	100 (90–100)	70 (40–95)	0.029
MRC after extubation	33 (24–40)	36 (30–40)	26 (16–40)	0.203
Rehabilitation after extubation (with assistance)				
Length of time until sitting, days	1 (1–2)	1 (1–2)	2 (1–3)	0.380
Length of time until standing, days	2 (1–5)	2 (1–3)	4 (1–7)	0.631
Length of time until ambulation, days	4 (3–10)	4 (3–9)	6 (2–14)	0.787
Laboratory data				
LDH (U/L)	568 (466–682)	593 (464–698)	535 (488–611)	0.896
CRP (mg/dL)	9.0 (4.8–12.8)	6.6 (3.7–10.4)	12.7 (9.3–13.7)	0.140
Hb (g/dL)	12.5 (10.9–13.5)	12.6 (10.7–13.3)	12.4 (11.4–13.5)	0.531
Alb (g/dL)	2.2 (2.1–2.3)	2.3 (2.1–2.4)	2.1 (2.0–2.2)	0.104
Frailty at hospital discharge, n (%)	18 (81.8)	10 (76.9)	8 (88.9)	0.471
Discharged to home, n (%)	13 (59.1)	12 (92.3)	3 (33.3)	0.006

## Discussion

This is the first study to investigate the effects of first-session prone positioning on short-term outcomes in critically ill patients with COVID-19 who required ventilator management. We found that responders had significantly shorter intubation periods, as well as higher BI scores and higher home discharge rates. This suggests that short-term outcomes can be predicted from an earlier stage and thus can guide treatment and physiotherapy.

Responders showed a significantly shortened duration of intubation. The improvement in oxygenation upon return from prone to a supine position may have resulted in effective correction of the ventilation-perfusion ratio mismatch. Additionally, compared with responders, non-responders showed a higher proportion of patients with pre-existing chronic respiratory disease (7.7% vs. 66.7%, p = 0.003). Accordingly, compared with responders, non-responders may have shown a worse response to prone positioning with respect to improvements in gas exchange capacity and ventilation-perfusion ratio mismatch due to organic problems, including reduced lung compliance and impaired diffusion.

Notably, obesity tended to be more prevalent among responders than among non-responders (53.8% vs. 11.1%, p = 0.074), which is consistent with a previous report on patients with obesity and ARDS [18]. Since the prone position is effective in gravity-dependent alveolar collapse, the release of pressure from the abdominal organs is more pronounced in patients with obesity. Moreover, it may ameliorate the decrease in functional residual capacity caused by increased abdominal pressure [18]. Although obesity is a risk factor for severe COVID-19 [19], patients with severe COVID-19 who have obesity may benefit from prone therapy as the preferred positioning strategy. Other studies have reported no evidence of efficacy [20], and understanding COVID-19-specific symptoms and sample size requires future research.

The disease course of severe COVID-19 is considered secondary to a systemic hyperinflammatory “cytokine storm” rather than reflective of lung damage directly caused by the virus [21]. Furthermore, muscle protein imbalance occurs when critically ill patients are forced to take to bed rest [22]. Excessive release of inflammatory cytokines and stress hormones leads to decreased protein synthetic capacity and increased degradation, which decreases muscle mass [23,24]. In our study, the mean total MRC score after extubation was 33 points, which was lower than the cut-off value for ICU-acquired weakness (48 points) [25], with no significant between-group difference (36 vs. 26 points, p = 0.203) (Table 3). A previous study reported that 70% of patients had limb muscle weakness after extubation [26], which is a lower incidence than that in our study. Mobilization and electrical muscle stimulation under intubation performed in this previous study may have led to better results than those in the present study. The accumulated doses of sedatives and muscle relaxants vary according to the duration of intubation. Large doses can cause hemodynamic instability [27] and rapid development of myopathy [22]. Therefore, their prolonged use may adversely influence ADLs after extubation. The shorter duration of intubation among responders may have minimized the accumulation of sedatives as well as the deterioration in respiratory and limb muscle strength. Accordingly, this allowed a faster recovery in ADLs and better outcomes than those in non-responders. Systemic inflammation, hypoxemia, prolonged bed rest, and extensive medications in critically ill patients with COVID-19 can cause muscle weakness, fatigue, and decreased exercise tolerance [28]. Therefore, low-impact, high-frequency physical therapy interventions in a confined space within the red zone are recommended early after extubation.

This study has several limitations. First, this was a single-center, small-scale retrospective study. The sample size was limited by our study design, given the exclusion of 84.2% (139/165) of non-ventilated patients with COVID-19 admitted to our hospital. Therefore, this may have an effect on the statistical results. Second, genomic analysis of SARS-CoV-2 viruses was unavailable. The study period overlapped with the peak of the prevalence of the delta strain in our country [29]. Since different mutant variants have different severities and pathologies, the clinical outcomes of patients may vary according to the mutant variant. Finally, we did not measure cytokine levels among patients with severe COVID-19 since the required facilities were unavailable at our center. Collaboration with specialized facilities should be pursued in the future. Extensive prevention of severe disease leads to better outcomes; therefore, further research is warranted to inform the prevention of severe disease in the moderate stage [7,30]. It is imperative to re-examine the type of physiotherapy intervention administered during intubation and perform objective assessments within the red zone.

## Conclusions

This study provides new information on the effects of first-session prone positioning on short-term outcomes in patients with severe COVID-19 requiring ventilator management. We found that improved response to the first-session prone positioning was related to better ADLs and outcomes at discharge in patients with severe COVID-19. Therefore, the prognosis of treatment and rehabilitation in patients with severe COVID-19 may be predicted at an early treatment stage. Further research is needed to confirm these results in patients with different mutant strains of SARS-CoV-2 and elucidate the role of cytokine levels in the relationships between responsiveness to first-session prone positioning and short-term outcomes.
